# Supplementary material for: Single-shot stereo-polarimetric compressed ultrafast photography for light-speed observation of high-dimensional optical transients with picosecond resolution
Source: Nat Commun. 2020 Oct 16;11:5252. doi: 10.1038/s41467-020-19065-5 (PMC7567836; doi:10.1038/s41467-020-19065-5)
Supplement: Supplementary file 1 — Supplementary Information [file 41467_2020_19065_MOESM1_ESM.pdf]

SUPPLEMENTARY MATERIALS

Single-shot stereo-polarimetric compressed ultrafast photography for light-speed observation of high-dimensional optical transients with picosecond resolution

Jinyang Liang[1,†,‡], Peng Wang[1,‡], Liren Zhu[1], and Lihong V. Wang[1,*]

[1]Caltech Optical Imaging Laboratory, Andrew and Peggy Cherng Department of Medical Engineering, Department of Electrical Engineering, California Institute of Technology, 1200 East California Boulevard, Mail Code 138-78, Pasadena, CA 91125, USA

[†]Present address: Laboratory of Applied Computational Imaging, Centre Énergie Matériaux Télécommunications, Institut National de la Recherche Scientifique, 1650 boulevard Lionel-Boulet, Varennes, QC J3X1S2, CANADA

*Corresponding author: LVW@caltech.edu

[‡]These authors contributed equally to this work

Supplementary Note 1. Operating principle of a streak camera

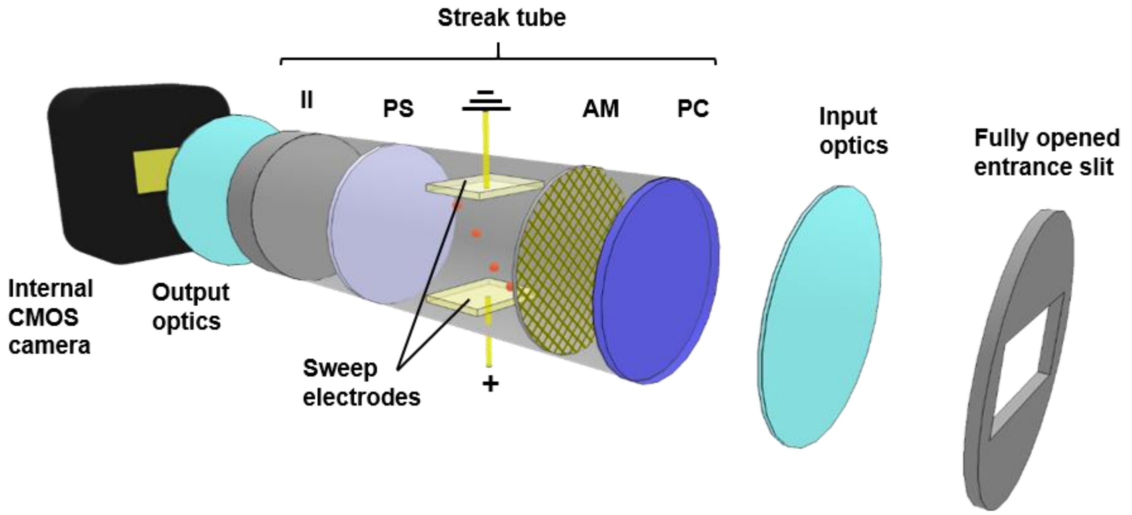

Supplementary Figure 1. Detailed illustration of the operating principle of the streak camera. AM, accelerating mesh; II, image intensifier; PC, photocathode; PS, phosphor screen.

Supplementary Note 2. SP-CUP's model of data acquisition

SP-CUP's data acquisition is schematically illustrated in Supplementary Figure 2. For generality, we do not consider the polarization sensing (denoted by operators $\boldsymbol{P_0}$ and $\boldsymbol{P_{45}}$) and the stereoscopic sensing (denoted by operators $\boldsymbol{Z_L}$ and $\mathbf{Z_R}$) in this derivation. The model of data acquisition in Views 1, 3, and 4 have been presented in a previous publication [1]. In the following, we derive the explicit expressions for Views 2, 5, and 6. For simplicity without losing generality, we make the following two assumptions. First, the imaging system has a unit magnification. Second, the external CCD camera and the internal CMOS camera of the streak camera have the same pixel size, denoted as d. To accommodate the streak camera's setting and simplify the notation, we choose a voxel of (d, d, τ_s) in size, in the x-y-t space, where $\tau_s = d/v$, and v denotes the temporal shearing speed. In the following, we first derive the expression of SP-CUP's data acquisition process in the continuous model and then discretize it for the image reconstruction algorithm.

In the second time-unsheared view (View 2), the input scene is first physically rotated by the dove prism,

$$I_{R_\mathrm{p}}(x,y,t) = \boldsymbol{R}_\mathbf{p}\{I(x,y,t)\} = I(-x,-y,t)\,, \tag{1}$$

where $\boldsymbol{R}_\mathbf{p}$ represents the 180° physical image rotation induced by the dove prism. Second, the scene is spatially low-pass filtered by the external CCD's camera lens,

$$I_{F_2}(x,y,t) = \boldsymbol{F_2}\left\{I_{R_\mathrm{p}}(x,y,t)\right\}, \tag{2}$$

where $\boldsymbol{F_2}$ represents the spatial low-pass filtering operator.

Next, an image distortion operator, $\boldsymbol{D_2}$, is applied to $I_{F_2}(x,y,t)$,

$$I_{D_2}(x,y,t) = \boldsymbol{D_2}\{I_{F_2}(x,y,t)\}\,. \tag{3}$$

Then, $I_{D_2}(x,y,t)$ is measured by the external CCD camera by

$$\begin{aligned} E_{2\mathrm{T}}[m,n] &= \boldsymbol{T}\{I_{D_2}(x,y,t)\} \\ &= \int dx \int dy \left\{\left[\int dt\, I_{D_2}(x,y,t)\right] \cdot \mathrm{rect}\left[\frac{x}{d} - \left(m + \frac{1}{2}\right), \frac{y}{d} - \left(n + \frac{1}{2}\right)\right]\right\}, \end{aligned} \tag{4}$$

where $\boldsymbol{T}$ represents spatiotemporal integration.

Finally, we digitally rotate $E_{2\mathrm{T}}[m,n]$ by 180°.

$$E_2[m,n] = \boldsymbol{R}_\mathbf{d}\{E_{2\mathrm{T}}[m,n]\} = E_{2\mathrm{T}}[-m,-n]\,, \tag{5}$$

where $\boldsymbol{R}_{\mathbf{d}}$ represents the 180° digital image rotation conducted by the external CCD camera and the streak camera.

Thus, the measured optical energy distribution at the second time-unsheared view is given by

$$E_2[m,n] = \int dx \int dy \left\{ \left[\int dt \, I_{D_2}(x,y,t) \right] \cdot \mathrm{rect}\left[\frac{x}{d} + \left(m - \frac{1}{2} \right), \frac{y}{d} + \left(n - \frac{1}{2} \right) \right] \right\}. \quad (6)$$

Supplementary Equation 6 indicates that the value in pixel $[m,n]$ in E_2 will be equal to that of pixel $[-m,-n]$ in the discretized $I_{D_2}(x,y,t)$.

For View 5, the dynamic scene is physically rotated by 180° (Supplementary Equation 1). Then, the intensity distribution of the dynamic scene is spatially encoded by a pseudo-random binary pattern displayed on a digital micromirror device (DMD), $c_{[10]}(x,y)$, yielding the following intensity distribution,

$$I_{C_5}(x,y,t) = c_{[10]}(x,y) I_{\boldsymbol{R}_{\mathbf{p}}}(x,y,t) \, . \quad (7)$$

Next, the spatially encoded dynamic scene passes through the imaging system to the entrance port of the streak camera. It is spatially low-pass filtered by the $4f$ imaging system between the DMD and the streak camera:

$$I_{F_5}(x,y,t) = \boldsymbol{F}_{\mathbf{5}}\{I_{C_5}(x,y,t)\} \, , \quad (8)$$

where $\boldsymbol{F}_{\mathbf{5}}$ denotes the spatial low-pass filtering operator in this time-sheared view.

Then, an image distortion operator is applied to $I_{F_5}(x,y,t)$,

$$I_{D_5}(x,y,t) = \boldsymbol{D}_{\mathbf{5}} \, \{I_{F_5}(x,y,t)\} \, , \quad (9)$$

where $\boldsymbol{D}_{\mathbf{5}}$ is due to image distortion from primarily the encoding arm and secondarily system misalignment. In stereoscopy, $\boldsymbol{D}_{\mathbf{5}}$ also includes distortion of the imaging lens $\boldsymbol{D}_{\mathbf{2}}$.

Next, the dynamic scene is captured by the streak camera. The temporal shearing along the vertical spatial axis can be expressed by

$$I_{S_5}(x,y,t) = \boldsymbol{S}\{I_{D_5}(x,y,t)\} = I_{D_5}(x, y - vt, t) \, . \quad (10)$$

As the next step, $I_{S_5}(x,y,t)$ is imaged by an internal CMOS camera in the streak camera. Akin to the time-unsheared view, the optical energy measured by the $[m,n]$ pixel on the CMOS takes the form

$$E_{5\mathrm{T}}[m,n] = \int dx \int dy \left\{ \left[\int dt \, I_{S_5}(x,y,t) \right] \cdot \mathrm{rect}\left[\frac{x}{d} - \left(m + \frac{1}{2} \right), \frac{y}{d} - \left(n + \frac{1}{2} \right) \right] \right\}. \quad (11)$$

Finally, we digitally rotate $E_{5\mathrm{T}}[m,n]$ by 180°,

$$E_5[m,n] = \boldsymbol{R}_{\mathbf{d}}\{E_{5\mathrm{T}}[m,n]\} = E_{5\mathrm{T}}[-m,-n]\,. \tag{12}$$

The optical energy distribution of the third time-sheared view (i.e., View 5) is given by

$$E_5[m,n] = \int dx \int dy \left\{\left[\int dt\, I_{S_5}(x,y,t)\right] \cdot \mathrm{rect}\left[\frac{x}{d} + \left(m - \frac{1}{2}\right), \frac{y}{d} + \left(n - \frac{1}{2}\right)\right]\right\}. \tag{13}$$

Similarly, we can derive the expression of the fourth time-sheared view (i.e., View 6)

$$E_6[m,n] = \int dx \int dy \left\{\left[\int dt\, I_{S_6}(x,y,t)\right] \cdot \mathrm{rect}\left[\frac{x}{d} + \left(m - \frac{1}{2}\right), \frac{y}{d} + \left(n - \frac{1}{2}\right)\right]\right\}. \tag{14}$$

To use this forward model in a compressed sensing-based reconstruction algorithm, we need to further discretize the dynamic scene to derive a discrete-to-discrete model. The discrete form of Views 1, 3, and 4 have been described in our previous publication [1]. Here we derive the discrete form for Views 2, 5, and 6.

The discrete form of the dynamic scene can be expressed as

$$I[m,n,k] = \int dt \int dx \int dy\, I(x,y,t) \cdot \mathrm{rect}\left[\frac{x}{d} - \left(m + \frac{1}{2}\right), \frac{y}{d} - \left(n + \frac{1}{2}\right), \frac{t}{\tau_s} - \left(k + \frac{1}{2}\right)\right]. \tag{15}$$

The measurement of View 2 can now be approximated by the following discrete form

$$E_2[m,n] = \frac{d^3}{v} \sum_k (h_2 * I)\left[f_{D_2}, g_{D_2}, k\right]. \tag{16}$$

Here, h_2 is the discrete convolution kernel of the operator $\boldsymbol{F}_2$, while f_{D_2} and g_{D_2} are the discrete coordinates transformed according to the operator $\boldsymbol{D}_2$. Given the calibrated 2D projection transformation defined by a 3-by-3 matrix,

$$\begin{bmatrix} a_{11} & a_{12} & a_{13} \\ a_{21} & a_{22} & a_{23} \\ a_{31} & a_{32} & 1 \end{bmatrix}, \tag{17}$$

one can express the coordinates f_{D_2} and g_{D_2} explicitly as

$$f_{D_2} = \mathrm{round}\left(\frac{a_{11}m + a_{12}n + a_{13}}{a_{31}m + a_{32}n + 1}\right), \tag{18.1}$$

and

$$g_{D_2} = \mathrm{round}\left(\frac{a_{21}m + a_{22}n + a_{23}}{a_{31}m + a_{32}n + 1}\right). \tag{18.2}$$

For View 5, the encoding mask is discretized to be $c_{[10]}[m,n]$, and we can express the encoded dynamic scene in discrete form as

$$I_{C_5}[m,n,k] = c_{[10]}[m,n] \cdot I_{R_p}[m,n,k]\,. \tag{19}$$

Therefore, the discretized form of View 5 can be represented by

$$E_5[m,n] = \frac{d^3}{v}\sum_k \big(h_5 * I_{C_5}\big)\big[f_{D_5}, g_{D_5} - k, k\big]\,. \tag{20}$$

Following the same procedure of Supplementary Equations 15–20, the discretized form of View 6 can be derived:

$$E_6[m,n] = \frac{d^3}{v}\sum_k \big(h_6 * I_{C_6}\big)\big[f_{D_6}, g_{D_6} - k, k\big]\,. \tag{21}$$

In summary, the measured optical energy distributions of Views 1–6 are linked to the dynamic scene by

$$\begin{aligned}
E_1[m,n] &= \frac{d^3}{v}\sum_k (h_1 * I)[m,n,k]\,,\\
E_2[m,n] &= \frac{d^3}{v}\sum_k (h_2 * I)\big[f_{D_2}, g_{D_2}, k\big]\,,\\
E_3[m,n] &= \frac{d^3}{v}\sum_k \big(h_3 * I_{C_3}\big)\big[f_{D_3}, g_{D_3} + k, k\big]\,,\\
E_4[m,n] &= \frac{d^3}{v}\sum_k \big(h_4 * I_{C_4}\big)\big[f_{D_4}, g_{D_4} + k, k\big]\,,\\
E_5[m,n] &= \frac{d^3}{v}\sum_k \big(h_5 * I_{C_5}\big)\big[f_{D_5}, g_{D_5} - k, k\big]\,,\\
E_6[m,n] &= \frac{d^3}{v}\sum_k \big(h_6 * I_{C_6}\big)\big[f_{D_6}, g_{D_6} - k, k\big]\,.
\end{aligned} \tag{22}$$

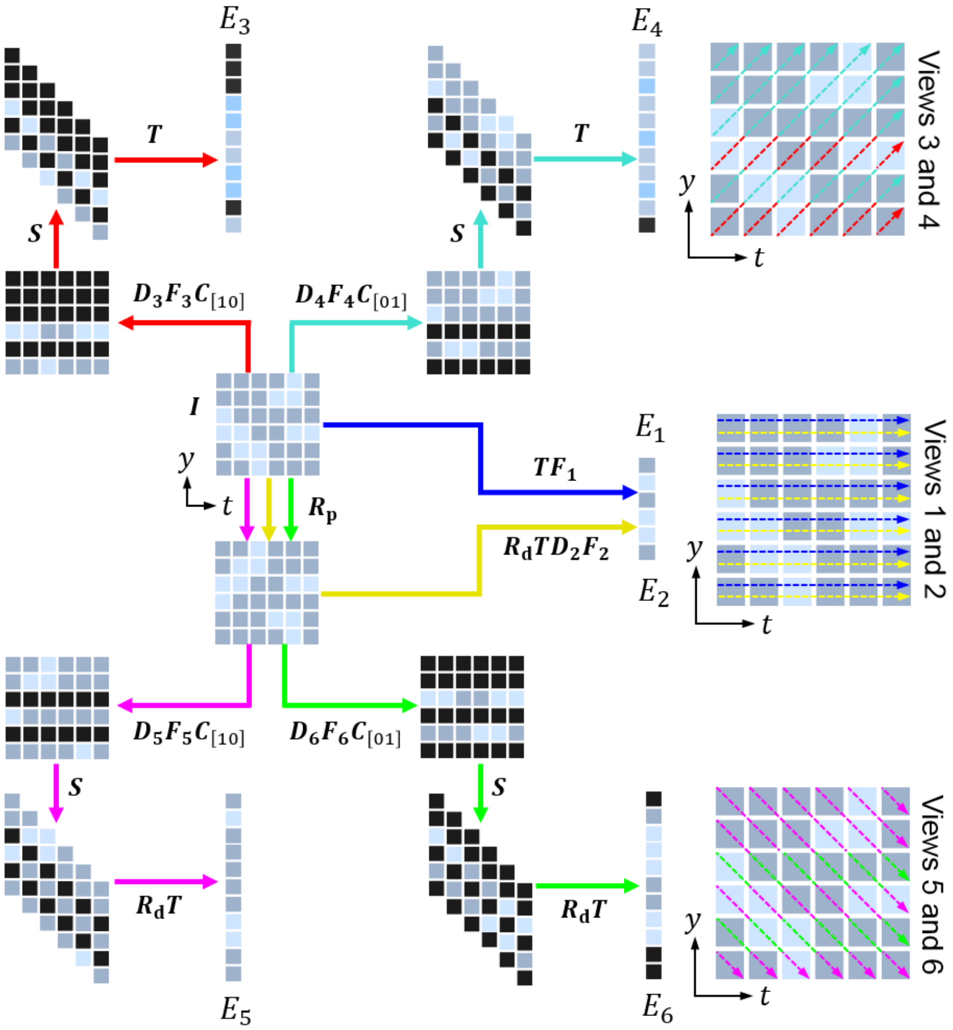

Supplementary Figure 2. Multi-view projection in SP-CUP's data acquisition illustrated by using a y-t slice in a datacube. In the two time-unsheared views (Views 1 and 2), the projection direction is parallel with the t-axis (blue and yellow dashed lines). In the first two time-sheared views (Views 3 and 4), the datacube is first temporally sheared and then spatiotemporally integrated. It is equivalent to directly spatiotemporally integrating the original datacube in a tilted direction that is not parallel with the t-axis (red and cyan dashed lines). Similarly, the other two time-sheared views (Views 5 and 6) will provide another projection view. However, because of the image rotation provided by the dove prisms, the equivalent projection direction is opposite (magenta and green dashed lines) to that of Views 3 and 4.

Supplementary Note 3. Details on SP-CUP's image reconstruction

Calibration of spatial encoding operator $\boldsymbol{C}$: Pre-processing the calibration image of the encoding mask is summarized in Supplementary Figure 3. First, background subtraction and white-field correction were performed. A reference image of the DMD code of all '1's was used to do white-field correction [2,3]. Then, several binarization methods were tried to generate the binary encoding mask for reconstruction. It was found that the binarization based on the combination of threshold selection and edge detection led to the best result.

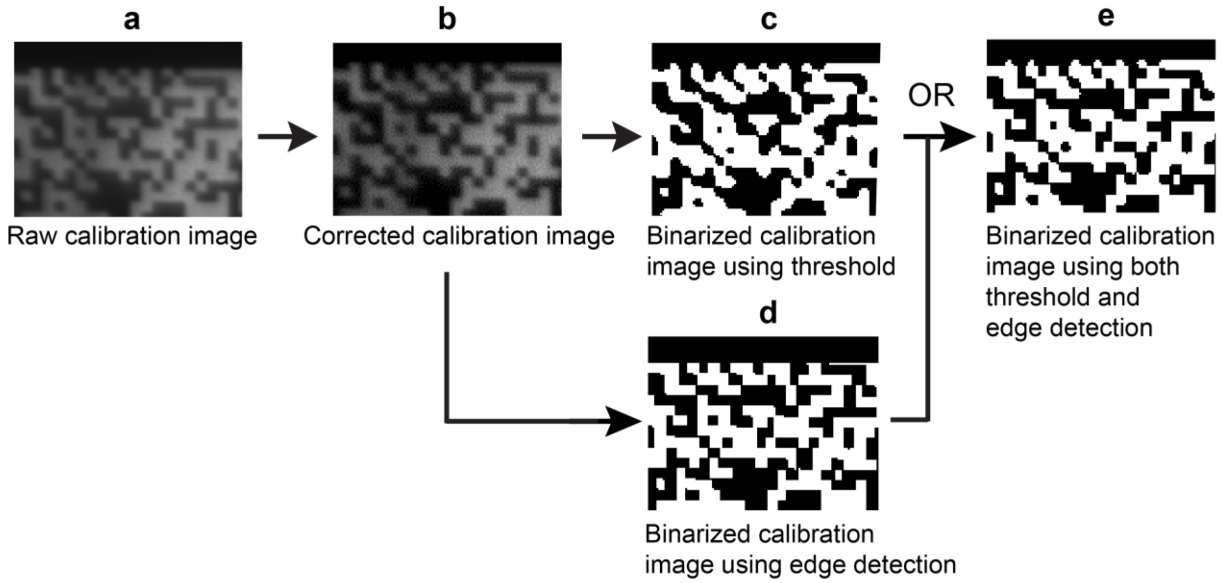

Supplementary Figure 3. Pre-processing of the calibration image of the encoding mask. (**a**), A section cropped from the raw calibration image. (**b**), After background subtraction and white-field correction. (**c**), Image binarization by applying a threshold to (**b**). (**d**)**,** Image binarization by detecting edges in (**b**). (**e**), After combining (**c**) and (**d**) using OR operation.

Calibration of the distortion operators $\boldsymbol{D_i}$: The distortion operators of all six raw views are $\boldsymbol{D_i}$ $(i = 1, \ldots, 6)$, in which $\boldsymbol{D_5} = \boldsymbol{D_3}\boldsymbol{D_2}$ and $\boldsymbol{D_6} = \boldsymbol{D_4}\boldsymbol{D_2}$. $\boldsymbol{D_3}$ and $\boldsymbol{D_4}$ were calibrated first. $\boldsymbol{D_3}$ was applied to Views 3 and 5, while $\boldsymbol{D_4}$ was applied to Views 4 and 6. Later on, the correction of $\boldsymbol{D_2}$ was carried out for Views 5 and 6. Here, $\boldsymbol{D_2}$ represents distortion in the left image in stereoscopy with respect to the right image (i.e., $\boldsymbol{D_1} = \boldsymbol{1}$). See Supplementary Note 9 for more details on $\boldsymbol{D_2}$ calibration.

Settings for the TwIST algorithm: Parameters were optimized for our reconstruction algorithm, developed from the TwIST algorithm [4], to achieve successful and accurate reconstruction. Supplementary Table 1 summarizes those important parameters used. These optimized parameters allowed us to achieve 94.58% in point cloud completeness, which describes the number of reconstructed points over the total number of object points [5].

Supplementary Table 1. Summary of important parameters used in image reconstruction.

Regularization parameter	*Initial solution*	*Regularizer*	*Maximum number of iterations*
10^{-4}	Pseudo-inverse	Total variation (TV) in (x, y, t) space	200

Supplementary Note 4. Summary of experimental settings

Arrangement of the polarizers: Supplementary Table 2 summarizes the angles of the polarizers used in each view in the SP-CUP system for the four experiments.

Supplementary Table 2. Summary of the arrangement of polarizers used for each experiment.

View / *Experiment*	1	2	3	4	5	6
Laser pulse sweeping across five letters	0°	45°	0°	0°	45°	45°
Plasma emission in LIB	0°	45°	0°	NoP	45°	NoP
Laser pulse sweeping across three shapes	0°	45°	0°	0°	45°	45°
Laser pulse propagating in a 3D scattering medium	0°	NoP	0°	0°	NoP	NoP

(3D: three-dimensional; LIB: laser-induced breakdown; NoP: No polarizer.)

Data acquisition: Supplementary Table 3 summarizes the forward model for each experiment. They are extensions of Equation 1 in Methods.

Supplementary Table 3. Summary of the forward model used for each experiment.

Laser pulse sweeping across five letters	Plasma emission in LIB
$\begin{bmatrix} E_1 \\ E_3 \\ E_4 \end{bmatrix} = \begin{bmatrix} \boldsymbol{TF_1} \\ \boldsymbol{TSD_3F_3C}_{[10]} \\ \boldsymbol{TSD_4F_4C}_{[01]} \end{bmatrix} \boldsymbol{P_0}\, I(x,y,t,\psi)$ and $\begin{bmatrix} E_2 \\ E_5 \\ E_6 \end{bmatrix} = \begin{bmatrix} \boldsymbol{R}_{\mathbf{d}}\boldsymbol{TF_2R}_{\mathbf{p}} \\ \boldsymbol{R}_{\mathbf{d}}\boldsymbol{TSD_3F_5C}_{[10]}\boldsymbol{R}_{\mathbf{p}} \\ \boldsymbol{R}_{\mathbf{d}}\boldsymbol{TSD_4F_6C}_{[01]}\boldsymbol{R}_{\mathbf{p}} \end{bmatrix} \boldsymbol{D_2P_{45}}\, I(x,y,t,\psi)$	$\begin{bmatrix} E_1 \\ E_3 \end{bmatrix} = \begin{bmatrix} \boldsymbol{TF_1} \\ \boldsymbol{TSD_3F_3C}_{[10]} \end{bmatrix} \boldsymbol{P_0}\, I(x,y,t,\psi)$ $\begin{bmatrix} E_2 \\ E_5 \end{bmatrix} = \begin{bmatrix} \boldsymbol{R}_{\mathbf{d}}\boldsymbol{TF_2R}_{\mathbf{p}} \\ \boldsymbol{R}_{\mathbf{d}}\boldsymbol{TSD_3F_5C}_{[10]}\boldsymbol{R}_{\mathbf{p}} \end{bmatrix} \boldsymbol{D_2P_{45}}\, I(x,y,t,\psi)$ $\begin{bmatrix} E_4 \\ E_6 \end{bmatrix} = \begin{bmatrix} \boldsymbol{TSD_4F_4C}_{[01]} \\ \boldsymbol{R}_{\mathbf{d}}\boldsymbol{TSD_2D_4F_6C}_{[01]}\boldsymbol{R}_{\mathbf{p}} \end{bmatrix} I(x,y,t,\psi)$
Laser pulse sweeping across three shapes	Laser pulse propagating in a 3D scattering medium
$\begin{bmatrix} E_1 \\ E_3 \\ E_4 \end{bmatrix} = \begin{bmatrix} \boldsymbol{TF_1} \\ \boldsymbol{TSD_3F_3C}_{[10]} \\ \boldsymbol{TSD_4F_4C}_{[01]} \end{bmatrix} \boldsymbol{P_0Z}_{\mathbf{R}}\, I(x,y,z,t,\psi)$ $\begin{bmatrix} E_2 \\ E_5 \\ E_6 \end{bmatrix} = \begin{bmatrix} \boldsymbol{R}_{\mathbf{d}}\boldsymbol{TF_2R}_{\mathbf{p}} \\ \boldsymbol{R}_{\mathbf{d}}\boldsymbol{TSD_3F_5C}_{[10]}\boldsymbol{R}_{\mathbf{p}} \\ \boldsymbol{R}_{\mathbf{d}}\boldsymbol{TSD_4F_6C}_{[01]}\boldsymbol{R}_{\mathbf{p}} \end{bmatrix} \boldsymbol{D_2P_{45}Z}_{\mathbf{L}}\, I(x,y,z,t,\psi)$	$\begin{bmatrix} E_1 \\ E_3 \\ E_4 \end{bmatrix} = \begin{bmatrix} \boldsymbol{TF_1} \\ \boldsymbol{TSD_3F_3C}_{[10]} \\ \boldsymbol{TSD_4F_4C}_{[01]} \end{bmatrix} \boldsymbol{P_0Z}_{\mathbf{R}}\, I(x,y,z,t,\psi)$ $\begin{bmatrix} E_2 \\ E_5 \\ E_6 \end{bmatrix} = \begin{bmatrix} \boldsymbol{R}_{\mathbf{d}}\boldsymbol{TF_2R}_{\mathbf{p}} \\ \boldsymbol{R}_{\mathbf{d}}\boldsymbol{TSD_3F_5C}_{[10]}\boldsymbol{R}_{\mathbf{p}} \\ \boldsymbol{R}_{\mathbf{d}}\boldsymbol{TSD_4F_6C}_{[01]}\boldsymbol{R}_{\mathbf{p}} \end{bmatrix} \boldsymbol{D_2Z}_{\mathbf{L}}\, I(x,y,z,t,\psi)$

Image reconstruction: In general, SP-CUP's image reconstruction includes two parts. The first part of the reconstruction was to reconstruct the spatiotemporal datacubes. The second part was to retrieve the depth (z) and the polarization states, based on the reconstructed spatiotemporal frames (Supplementary Table 4). Please see Supplementary Notes 6 and 9 for details.

Supplementary Table 4. Retrieval of depth and polarization states from the reconstructed spatiotemporal datacubes.

Laser pulse sweeping across five letters	Plasma emission in LIB
$I_0(x,y,t) = \boldsymbol{P_0}I(x,y,t,\psi)$<br>$I_{45}(x,y,t) = \boldsymbol{P_{45}}I(x,y,t,\psi)$	$I_0(x,y,t) = \boldsymbol{P_0}I(x,y,t,\psi)$,<br>$I_{45}(x,y,t) = \boldsymbol{P_{45}}I(x,y,t,\psi)$, and<br>$I_{\mathrm{NoP}}(x,y,t)$
Laser pulse sweeping across three shapes	Laser pulse propagating in a 3D scattering medium
$I_0^{\mathrm{R}}(x,y,t) = \boldsymbol{P_0}\boldsymbol{Z_{\mathrm{R}}}I(x,y,z,t,\psi)$, and<br>$I_{45}^{\mathrm{L}}(x,y,t) = \boldsymbol{P_{45}}\boldsymbol{Z_{\mathrm{L}}}I(x,y,z,t,\psi)$	$I_0^{\mathrm{R}}(x,y,t) = \boldsymbol{P_0}\boldsymbol{Z_{\mathrm{R}}}I(x,y,z,t,\psi)$, and<br>$I^{\mathrm{L}}(x,y,t) = \boldsymbol{Z_{\mathrm{L}}}I(x,y,z,t,\psi)$

Supplementary Note 5. Summary of general system characterizations

Experimental quantification of the spatial and temporal resolutions, the spatial and temporal ranges, and the signal-to-noise ratio (SNR): The spatial and temporal resolutions of the SP-CUP system have been quantified under each experimental condition. We recorded an (x, y, t) dynamic scene: Two laser pulses, each with a full-width-at-half-maximum pulse width of 0.5 ps, sequentially illuminated through a transmissive spoke pattern. We tuned the time delay between the two pulses to quantify the temporal resolution, which is defined by applying the Rayleigh criterion [6] in the time domain. To calculate the spatial resolution, we projected the (x, y, t) datacubes onto the x-y plane by summing over the voxels along the temporal axis. Then, these temporally integrated images were Fourier transformed for bandwidth analysis. The cutoff bandwidth was defined as when the power spectral density drops to two times the standard deviation of the background. The SNR of the captured raw data was calculated by dividing the peak intensity by the standard deviation of the background and then converting to dB. The results are summarized in Supplementary Table 5.

Supplementary Table 5. Experimentally quantified spatial and temporal resolutions and signal-to-noise ratio.

Parameter / *Experiment*	Imaging speed (Gfps)	Spatial resolution			Temporal resolution (ps)	Signal-to-noise ratio (dB)
		x	y	z		
Laser pulse sweeping across five letters	250	2.67 mm	4.44 mm	—	19	29
Plasma emission in LIB	100	5.80 μm	10.39 μm	—	41	28
Laser pulse sweeping across three shapes	100	0.82 mm	1.42 mm	5.5 mm	35	31
Laser pulse propagating in a 3D scattering medium	100	3.15 mm	5.46 mm	4.0 mm	35	26

Theoretical estimates of the spatial and temporal resolutions: The spatial resolutions in the x and y dimensions are mainly limited by the encoding pixel size of the spatial encoding mask, the SNR in the acquired data, and the overall magnification ratio of the SP-CUP system. First, to satisfy the Nyquist sampling theory, the minimum spatial feature must be sampled by at least two encoding

pixels. Moreover, due to the mixture in information in the y and t dimensions, the spatial resolution in the y dimension is worse than that in the x dimension [4,7]. The normalized resolutions can be further reduced if the acquired data have low SNRs. Finally, the encoding pixel size and the overall magnification ratio give the physical dimension of the spatial resolutions.

A similar analysis can estimate the resolution in the t dimension. In data acquisition, the information in the t dimension is mixed with that in the y dimension. Thus, the normalized resolutions (in terms of pixels) in the t and y dimensions are similar. In the t dimension, each encoding pixel corresponds to a certain time interval that is determined by the shearing speed. In this way, the temporal resolution can be determined.

Finally, the resolution in the z dimension in stereoscopy (detailed in Supplementary Note 9) is mainly limited by the SNR in the captured data. This SNR determines the resolvable disparity in the captured image sets. In our experiments, the normalized minimum disparity was ±0.1 pixels. Besides, other system parameters, such as the distance between two channels, the focal length of the imaging lens, the sensor's pixel size, and the distance to the scene, also affect the z resolution.

The estimated spatial and temporal resolutions are listed in Supplementary Table 6. They agree well with the measured resolutions.

Supplementary Table 6. Theoretically estimated spatial and temporal resolutions

Parameter / *Experiment*	Imaging speed (Gfps)	Encoding pixel size	Magnification ratio	Estimated spatial resolution			Estimated temporal resolution
				x	y	z	
Laser pulse sweeping across five letters	250	37.8 μm	0.018	2.10 mm	4.20 mm	—	12 ps
Plasma emission in LIB	100	37.8 μm	7.4	5.1 μm	10.2 μm	—	30 ps
Laser pulse sweeping across three shapes	100	37.8 μm	0.054	0.70 mm	1.40 mm	2.75 mm	30 ps
Laser pulse propagating in a 3D scattering medium	100	37.8 μm	0.014	2.70 mm	5.40 mm	2.00 mm	30 ps

The range of (x, y, t) is limited by the size of the internal CMOS sensor (with the numbers of pixels in columns and rows $N_c \times N_r$) in the streak camera. In the SP-CUP system, the sensor is

divided into $K = 4$ regions (see Fig. 1 in Main Text). Therefore, for the reconstructed datacube with the size of $N_x \times N_y \times N_t$, these numbers are related to N_c and N_r by $N_x \le N_\mathrm{c}/K$ and $N_y + N_t \le N_\mathrm{r}$. In the current SP-CUP system, we chose $N_x = 500$, $N_y = 600$, and $N_t = 300$ to balance the imaging field of view, sequence depth, and the speed of image reconstruction. In addition, the range of z is limited by the depth of field of the camera lens, which was 300 mm in our experiments.

Supplementary Note 6. Sensing light polarization using SP-CUP

Calculation of the polarization state of light: For any linearly polarized light, its electric field, E, can be written as

$$E = \left|\sqrt{I}\right| \times \begin{bmatrix} \cos\psi \\ \sin\psi \end{bmatrix}, \tag{23}$$

where I denotes intensity, and ψ represents the angle of linear polarization (AoLP).

In our experiments (see Sections 2 and 4 in Results), when the linearly polarized light passes through two polarizers whose polarization axes are positioned at 0° and 45°, the transmitted intensities are

$$I_0 = I \times (\cos\psi)^2 \text{, and} \tag{24}$$

$$I_{45} = \gamma \times \left({}^{I}\!/_{2}\right) \times (\cos\psi + \sin\psi)^2 \,. \tag{25}$$

Here, γ is an experimentally measured weighting factor to balance the transmission difference between two stereoscopic views. Therefore, the AoLP is calculated by

$$\psi = \tan^{-1}\left(\pm\sqrt{\frac{2 \times I_{45}}{\gamma \times I_0}} - 1 \right). \tag{26}$$

Here, the negative sign is used when both I_0 and I_{45} are smaller than a threshold, otherwise, the positive sign is used. The threshold is defined as half of the maximum imaged intensity in the time-unsheared view. Additionally, the first Stokes parameter—the light intensity from the object—is calculated by [8]

$$S_0 = I = \frac{I_0}{(\cos\psi)^2} = \frac{2 \times I_{45}}{\gamma \times (\cos\psi + \sin\psi)^2} \,. \tag{27}$$

Supplementary Equations 26 and 27 were used to calculate ψ and S_0 in both plano-polarimetric imaging of a laser pulse sweeping across five letters and stereo-polarimetric imaging of a laser pulse sweeping across three shapes.

In the plano-polarimetric imaging of laser-induced plasma (see Section 3 in Results), we assume the plasma emission consists of both randomly polarized light and linearly polarized light of unknown AoLP (ψ). Utilizing the configuration in the first table in Supplementary Note 4, we can characterize the emitted photons in all three parameters: S_0, ψ, and DoLP [8]:

$$S_0 = I_{\mathrm{NoP}} \,, \tag{28.1}$$

$$\psi = \frac{1}{2}\tan^{-1}\left(\frac{S_2}{S_1}\right), \text{ and} \tag{28.2}$$

$$\mathrm{DoLP} = \frac{\sqrt{S_1^2 + S_2^2}}{I_{\mathrm{NoP}}}\ , \tag{28.3}$$

where $S_1 = 2\gamma_0\eta I_0 - I_{\mathrm{NoP}}$ and $S_2 = 2\gamma_{45}\eta I_{45} - I_{\mathrm{NoP}}$. Here, γ_0 is a measured weighting factor that balances the transmission difference between the 0°-filtered view and the unfiltered view. γ_{45} is another one that balances the 45°-filtered view and the unfiltered view.

In the experiment imaging pulse propagation in a 3D scattering medium (see Section 5 in Results), the degree of linear polarization (DoLP) was calculated. Linearly polarized light was sent into the scattering medium and a linear polarizer with its transmission angle aligned with the incident light was applied in one of the stereoscopic views. In this experiment, we assume that the light scattered into the SP-CUP system is a mixture of both randomly polarized light and linearly polarized light with polarization in the direction aligned with that of the incident pulse. Therefore, DoLP was calculated by the intensity ratio between the view with a linear polarizer I_0 and the view with no polarizer I_{NoP} :

$$\mathrm{DoLP} = \gamma \times \frac{2 \times \sum_x \sum_y I_0}{\eta \times \sum_x \sum_y I_{\mathrm{NoP}}} - 1. \tag{29}$$

Here, η is the experimentally measured transmission efficiency of the linear polarizer.

<u>Quantification of the accuracy of measured DoLPs:</u> The experimental setup to quantify the accuracy of measured DoLP is shown in Supplementary Figure 4a. A 400-nm, 55-fs laser pulse passed through a polarizer to purify the pulse's linear polarization with the AoLP at 0°. Then, it was equally split by a beam splitter into two arms with the same length. The transmitted component passed through a quarter-wave plate to generate circularly polarized light. One tunable neutral density filter was inserted in each arm to equalize the ratio between the linearly polarized and circularly polarized light. Then, both arms were combined by another beam splitter to produce a DoLP of 0.5. Another variable neutral density filter was used to control the overall light intensity. Finally, the light passed through a negative USAF resolution target.

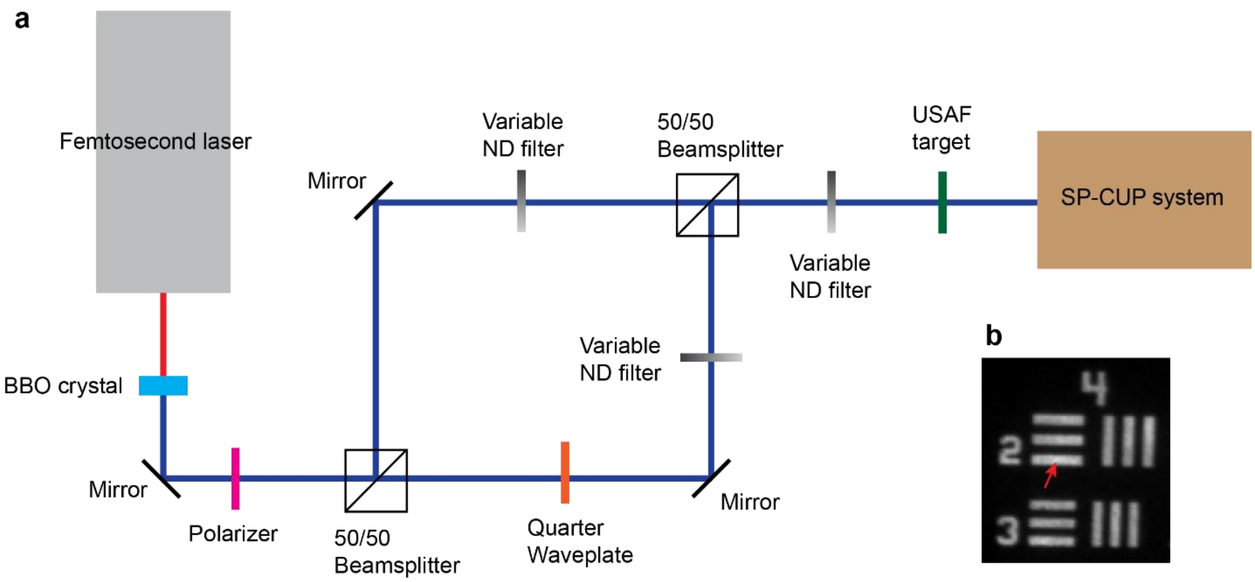

Supplementary Figure 4. Quantification of the accuracy of measured DoLPs. (**a**), Experimental setup. BBO, Beta barium borate; ND, neutral density. (**b**), Image of the USAF resolution target. The third horizontal bar in Element 2 of Group 4 (pointed by a red arrow) was used for the DoLP analysis.

We imaged this event using the SP-CUP system. Linear polarizers with its transmission angle aligned with that of the incident light were attached to Views 1, 3 and 4. No polarizers were attached to the other Views. The neutral density filter before the USAF resolution target was adjusted to preset the SNR in the raw data to 35, 30, 25, and 20 dB. After image reconstruction, we computed the mean value and standard deviation of the measured DoLP within the selected spatial feature (marked in Supplementary Figure 4b). The results are summarized in Supplementary Table 7.

Supplementary Table 7. Experimentally quantified accuracy of measured DoLPs under different SNRs.

Signal-to-noise ratio (dB)	*Mean measured DoLP*	*Standard derivation of measured DoLP*
35	0.50	0.022
30	0.48	0.031
25	0.47	0.044
20	0.55	0.060

These results show that SP-CUP can accurately measure the DoLP. For the SNRs of the experiments in our work (i.e., 26–31 dB, see Supplementary Table 5), the values measured by SP-CUP have an error of <5% compared to the preset value (i.e., 0.5). In addition, the measured DoLP values are tightly distributed around the ground truth, which demonstrates that SP-CUP can accurately sense DoLP over space.

The errors in the calculation of AoLP and DoLP: The errors in the calculation of AoLP and DoLP are mainly induced by the limited performance of the streak camera and by the reconstruction algorithm. The streak camera induces noise during the photon-to-photoelectron conversion in the photocathode, photoelectron-to-photon conversion in the phosphor screen, and the optical signal amplification inside the image intensifier. All these random noises cannot be easily calibrated. On the other hand, simply increasing the illumination intensity can induce the space-charge effect, which blurs the streak image. As a result, the SNR in the raw data is rather limited. This limited SNR is transferred to the reconstructed images. The reconstruction uses the prior knowledge of the pattern to distribute the intensity in the raw image to proper spatial and temporal voxels.

The errors in AoLP and DoLP could be mitigated by tackling these noise sources. For example, we need to carefully control the photon flux at the streak camera since too few photons reduce SNR while too many photons aggravate the space-charge effect. Furthermore, various paradigms of optical streaking [9,10] could be employed to avoid the photon-to-photoelectron conversion in the photocathode and the photoelectron-to-photon conversion in the phosphor screen. Finally, image reconstruction algorithms can be improved by developing different penalty terms and/or regularizers. Additional capabilities of noise elimination could be achieved by implementing advanced denoisers, such as the BM3D denoiser [11].

Supplementary Note 7. Additional results for plano-polarimetric ultrafast $(\boldsymbol{x}, \boldsymbol{y}, \boldsymbol{t}, \boldsymbol{\psi})$ imaging

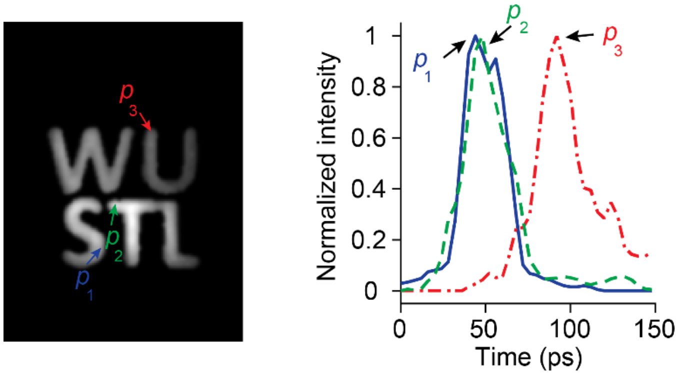

Supplementary Figure 5. Reconstructed intensity evolutions at three selected pixels $\boldsymbol{p_1}$, $\boldsymbol{p_2}$ and $\boldsymbol{p_3}$.

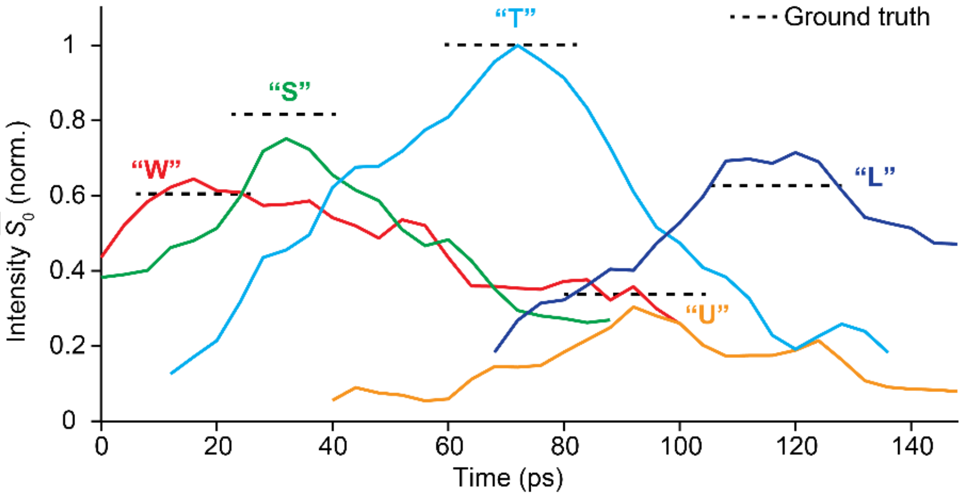

Supplementary Figure 6. Average $\boldsymbol{S_0}$ in five letters 'L', 'S', 'T', 'U', and 'W' versus time. The black dashed lines are the ground truths measured using the static time-unsheared images. Note that the finite lengths of these dashed lines are for illustration only and do not indicate durations of events.

Supplementary Note 8. Additional results on real-time polarization-resolved imaging of plasma emission in laser-induced breakdown

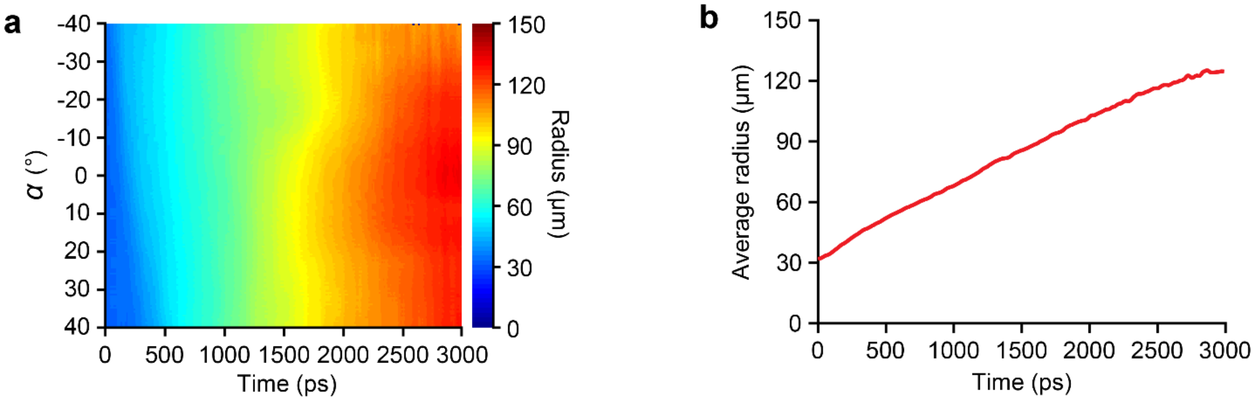

Supplementary Figure 7. Expansion of the plasma plume. (**a**), Evolution of radii of plasma plume front at different angles. (**b**), Evolution of the angularly averaged radius.

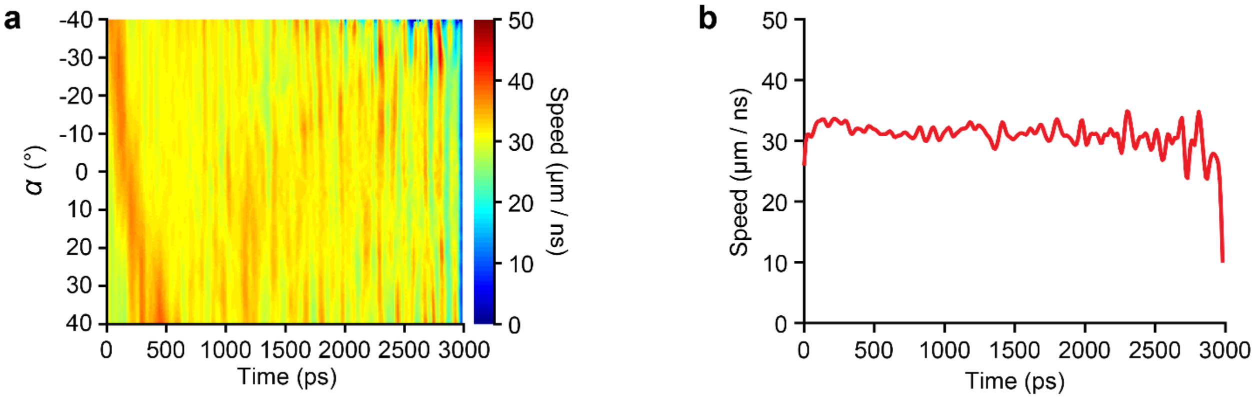

Supplementary Figure 8. Expansion speed of the plasma plume. (**a**), Instantaneous speed versus expansion angle and time. (**b**), Angularly averaged speed versus time.

The plasma emission from LIB is difficult to reproduce quantitatively because the nonlinear phenomenon is highly sensitive to the initial conditions such as the laser beam parameters and the wafer homogeneity [12]. This difficult-to-reproduce nature of LIB prevents the pump-probe method from accurately quantifying the critical parameters of interest in this process. To illustrate this point, we synthesized a 10-frame pump-probe measurement of plasma emission, shown in Supplementary Figure 9a. We repeated the measurement 10 times using the same experimental

setup and then used SP-CUP to reconstruct the movies. Shown in the top row of Supplementary Figure 9a, we stitched a pump-probe sequence by selecting one snapshot at a different time from each reconstruction (e.g., the snapshot at 1000 ps from the first reconstructed movie, the snapshot at 1100 ps from the second reconstructed movie, and so on). As a comparison, we show the result of SP-CUP from a single acquisition (see the bottom row of Supplementary Figure 9a). In stark contrast with the single-shot SP-CUP reconstruction, the pump-probe sequence displays severe random variations in the spatial profile of plasma emission (see Supplementary Figure 9a and Supplementary Movie 3).

Next, the plume fronts calculated based on the synthesized pump-probe and SP-CUP results are plotted in Supplementary Figures 9b and 9c, respectively. The SP-CUP's plots show uniform outward expansions, while those of the synthesized pump-probe method exhibit obvious irregularities. In linear regression of the plume front positions versus time, SP-CUP offers a goodness of fit, defined as the sum of the squared errors, as small as 6.7 μm^2, at all angles α, while the synthesized pump-probe method yields one greater than 100 μm^2, ~15× worse. Finally, we computed the plume's instantaneous expansion speeds at different angles α between –50° and 50° with a step of 0.5^o. The time-averaged speeds and their standard deviations are plotted in Supplementary Figure 9d. The synthesized pump-probe method and SP-CUP give SDs of 18 μm ns^{-1} and 2.2 μm ns^{-1}, respectively. This 8.2× degradation in SD suggests that averaging ~67 images at each time delay may be required for a pump-probe method to attain the same quantitative accuracy as SP-CUP in a single shot.

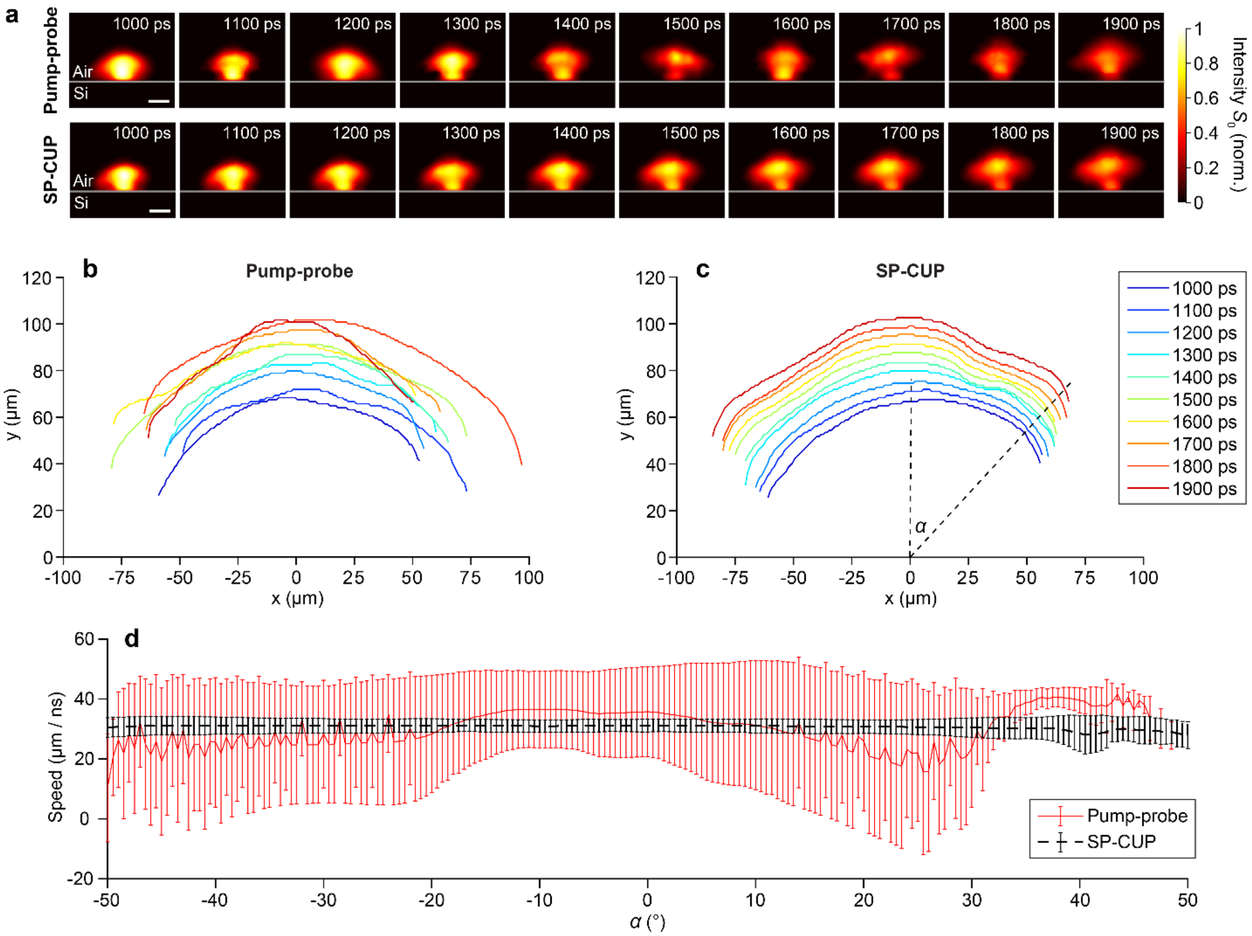

Supplementary Figure 9. Comparisons between the pump-probe method and SP-CUP in observing the plasma emission from LIB. (**a**), Snapshots of the normalized intensity dynamics of plasma emission. First row: pump-probe sequence synthesized by selecting frames from ten independent SP-CUP acquisitions. Second row: SP-CUP reconstruction from one single acquisition. The full sequences are included in Supplementary Movie 3. (**b**) and (**c**), positions of the expanding plasma plume fronts at 10 different time points from 1000 ps to 1900 ps, measured by the synthesized pump-probe method (**b**) and SP-CUP (**c**). (**d**), Average expansion speeds of the plume's front at different angles, α, defined in (**c**), quantified using the synthesized pump-probe method (red solid line) and SP-CUP (black dashed line). Scale bars in (**a**): 50 µm. Error bars in (**d**): standard deviations.

Supplementary Note 9. Principle, calibration, and distortion correction of stereoscopic measurements

Stereoscopy is widely used in machine vision for 3D profiling [13] [14]. The working principle of common-main-objective stereoscopy is shown in Supplementary Figure 10. Two identical imaging lenses of focal length *f* are separated by a distance of *l* on the baseline ($O_\mathrm{L}O_\mathrm{R}$). O_L and O_R are the optical centers of the lenses. P_L and P_R are the centers of the left and right images. Any point in 3D space (x, y, z) is projected to $(x_\mathrm{L}', y_\mathrm{L}')$ and $(x_\mathrm{R}', y_\mathrm{R}')$ in the left and right images, respectively. Due to parallax, the two acquired images (named left image and right image) are displaced from one another. The disparity between the two image points in the baseline direction (here, the horizontal direction) is $\delta = x_\mathrm{R}' - x_\mathrm{L}'$.

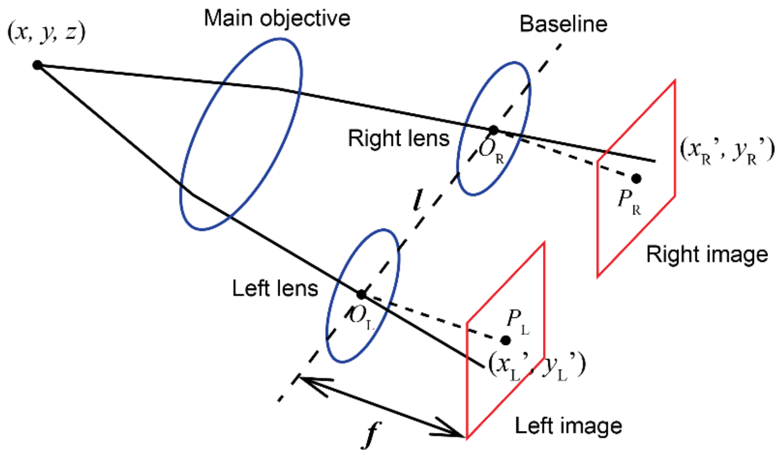

Supplementary Figure 10. Working principle of stereoscopy.

The purpose of calibration is to obtain the intrinsic, extrinsic, and distortion parameters of the stereoscopic camera, based on the spatial coordinates of a group of detected corners in N image pairs ($N > 3$, Supplementary Figure 11). Any point in the object space (x, y, z) is projected to image point (x', y') by [15]

$$\text{Constant} \times \begin{bmatrix} x' \\ y' \\ 1 \end{bmatrix} = \begin{bmatrix} f_x & s & c_x \\ 0 & f_y & c_y \\ 0 & 0 & 1 \end{bmatrix} \times [R \quad T] \times \begin{bmatrix} x \\ y \\ z \\ 1 \end{bmatrix}. \tag{30}$$

Here, f_x and f_y are the lens' focal lengths in the horizontal and vertical directions, s is the image skew, and c_x and c_y are the coordinates of the optical center. Matrices *R* and *T* are the rotation and translation transformations of the camera. As distortions are nonlinear, they are not included in

this expression. MATLAB Image Processing Toolbox was employed for fast and accurate calibration [16].

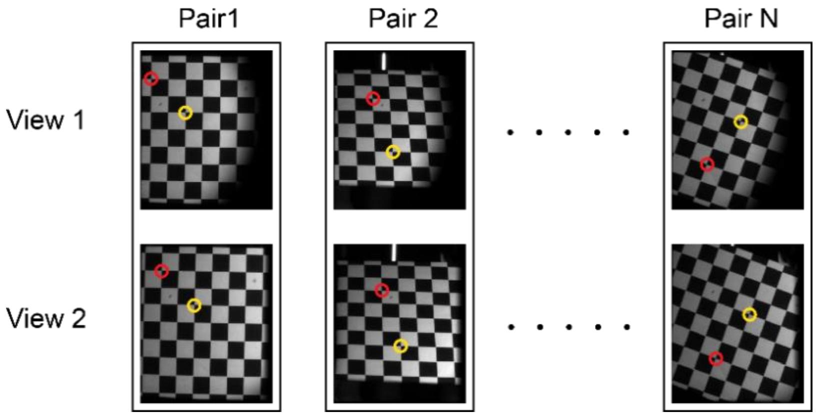

Supplementary Figure 11. Image pairs for stereoscopic camera calibration, captured by the external CCD camera (Views 1 and 2). A checkerboard of 10 mm squares was the target. In different image pairs, the checkerboard was placed at different positions and angles. Red and yellow circles represent paired corners between the image pairs.

The calibrated values of the key stereoscopic parameters used in this work were focal length f = 9.18 mm and lateral translation between the two cameras (i.e., the baseline separation) l = 8.56 mm. The pixel size is d = 3.69 µm. Then the depth z of any point is calculated by Equation 3 in Methods. When applying the semi-global block matching (SGBM) method [17] in disparity (δ) calculation, we chose an optimal block size of 17 pixels and a disparity range of (22, 38). SGBM works by calculating cross-correlations between the blocks with different spacings. In practice, it ends up with a small number of minor patches of pixels that are missing. Therefore, an algorithm was developed in house to detect these empty patches, and 2D linear interpolation was used to fill them in [18] based on the prior knowledge of continuously flat objects. An example of calculated disparity is shown in Supplementary Figure 12.

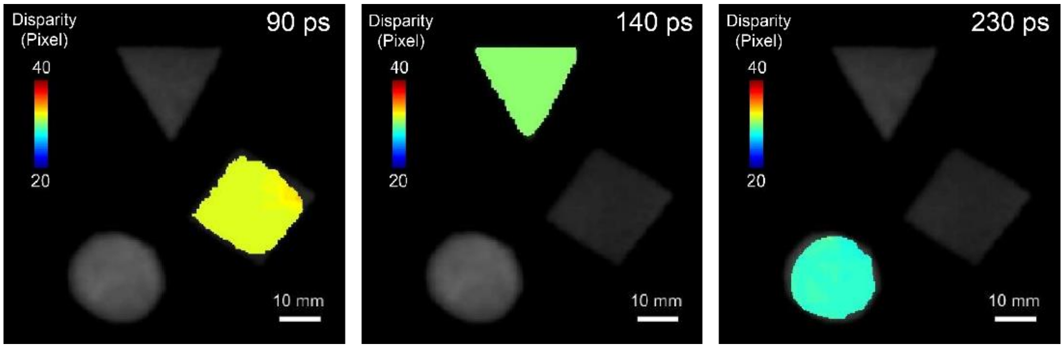

Supplementary Figure 12. Calculated disparity maps (δ) at three representative frames.

Correction of distortion $\boldsymbol{D_2}$ is important since the success of quantitative stereo imaging lies in the accuracy of pairing pixels in the left and right images that are from the same point in the object [19]. Sub-pixel accuracy is desired. In this work, both radial and tangential distortions were considered. One example is shown in Supplementary Figure 13. For plano-polarimetric ultrafast imaging (Figs. 2 and 3 in Main Text) that does not need stereo calibration, distortion can be simply compensated for by computing the affine transformation matrix between the two time-unsheared views (Views 1 and 2). Since the planar objects were placed at the same depth, two imaging channels experienced a fixed parallax. This constant parallax can be easily included in $\boldsymbol{D_2}$.

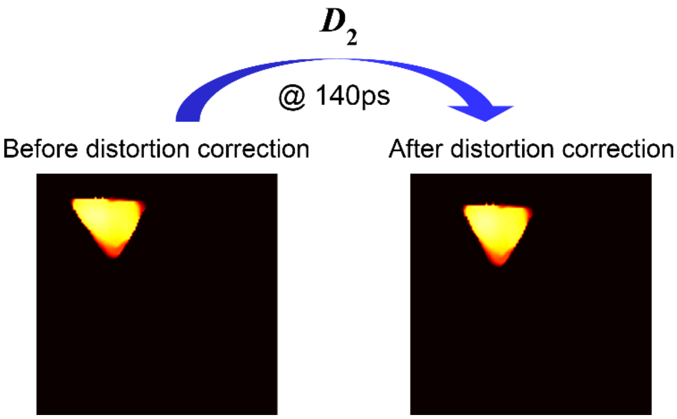

Supplementary Figure 13. Effect of distortion correction using the calibrated stereo camera parameters.

Supplementary Note 10. Additional results for stereo-polarimetric ultrafast $(\boldsymbol{x}, \boldsymbol{y}, \boldsymbol{z}, \boldsymbol{t}, \boldsymbol{\psi})$ *imaging*

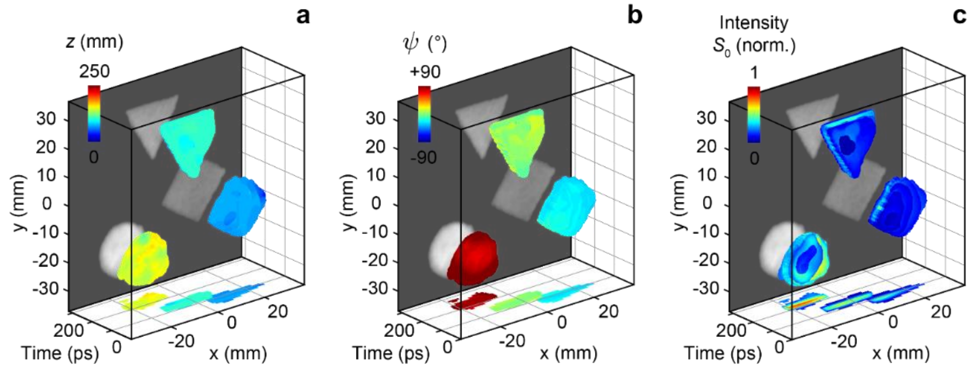

Supplementary Figure 14. Evolutions of the depth z (a), the AoLP ψ (b), and the first Stokes parameter S_0 (c) on the x-y plane. They are 4D subsets of the 5D data captured by the SP-CUP system. The backside of the plot is the time-unsheared image captured by the external CCD camera. The projections on the x-t plane are also shown.

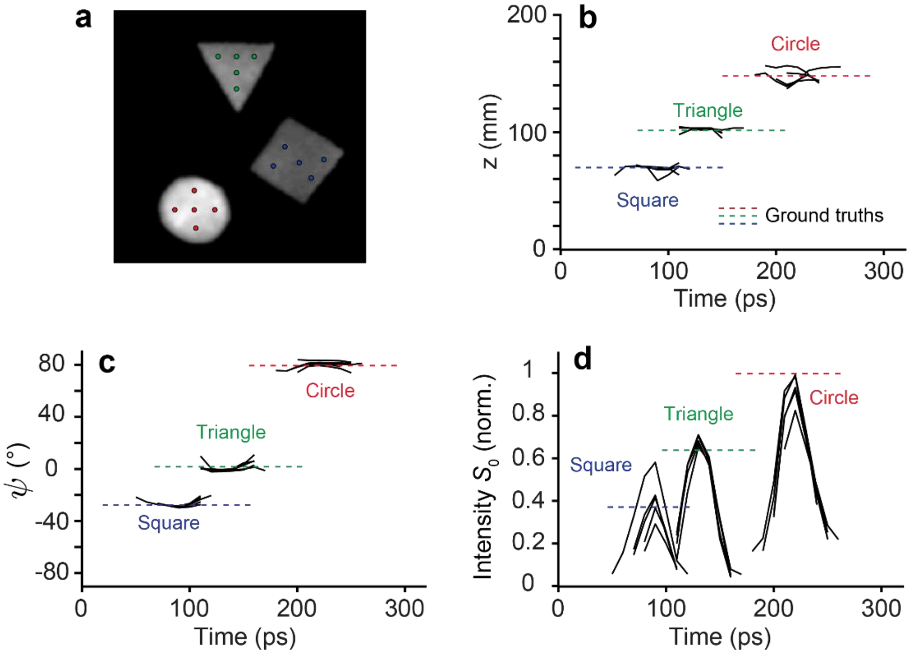

Supplementary Figure 15. (a), Static image of the objects using View 1. Five pixels in each shape are selected. Their reconstructed depth (b), AoLP ψ (c), and normalized S_0 (d) versus time are plotted. Black solid lines are the reconstructed data, and dashed lines are the ground truths measured by the static time-unsheared images. The finite lengths of these dashed lines are for illustration only and do not indicate durations of events.

Supplementary Note 11. Additional results for real-time imaging of pulse propagation in a 3D scattering medium

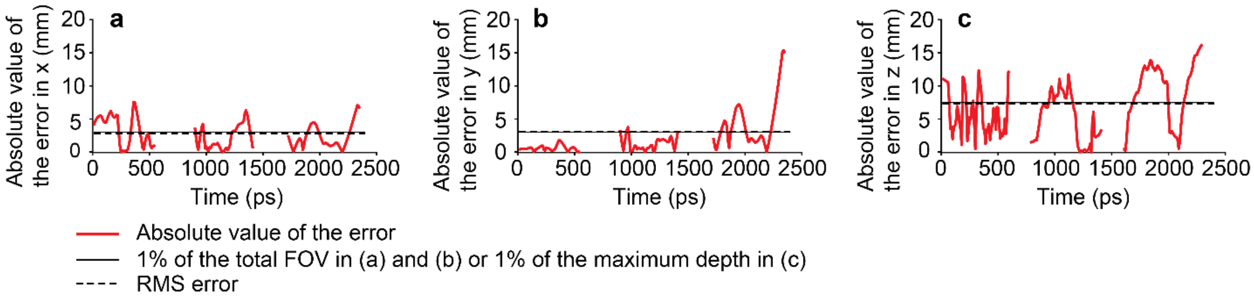

Supplementary Figure 16. Absolute values of the errors of the pulse centroids in three spatial dimensions. The black dashed lines represent the root-mean-square (RMS) errors. They are 2.82 mm, 3.10 mm, and 7.29 mm in x, y, and z directions, respectively. The black solid lines represent 1% of the field of view (FOV) in (**a**) and (**b**) and 1% of the maximum depth in (**c**).

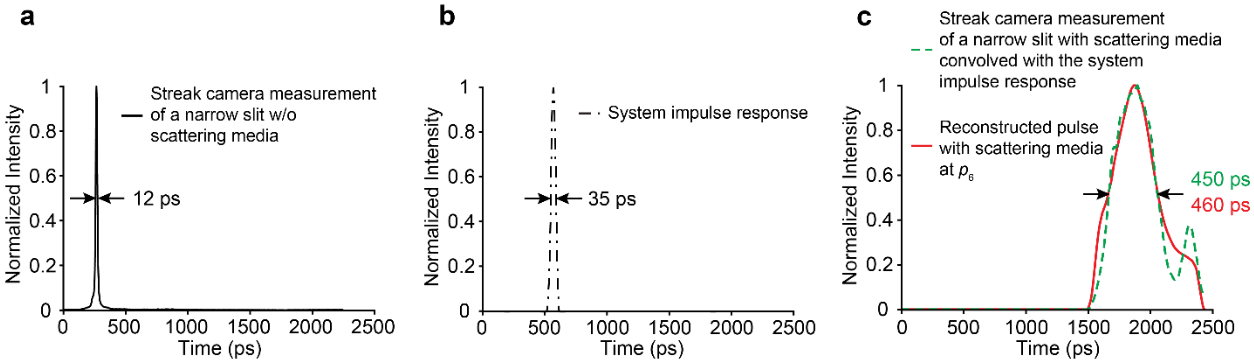

Supplementary Figure 17. Comparison between the reconstructed and the directly measured pulse intensity profile in the time domain. To measure the pulse duration, the streak camera was used to directly image an incident 55-fs pulse with a 40-µm-wide entrance slit at a one-dimensional imaging speed of 100 billion frames per second. (**a**), Intensity profile of the narrow slit when the pulse propagated without the scattering medium. (**b**), Reconstructed system impulse response of SP-CUP. (**c**), The green dashed line plots the imaged intensity evolution of the narrow slit illuminated by the pulse that propagated through the scattering medium, convolved with the system impulse response. The red solid line is the reconstructed pulse intensity profile right before the pulse exited the scattering medium. SP-CUP and direct streak camera imaging were carried out at different times, but the density of the scattering medium remained the same.

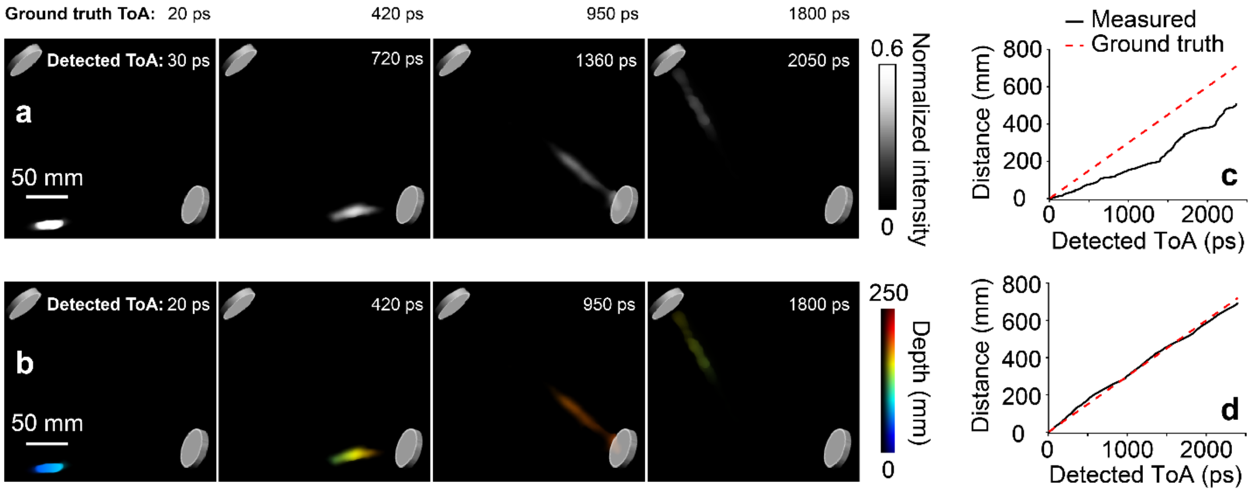

Supplementary Figure 18. Comparison of the results reconstructed by the previous CUP system and the newly developed SP-CUP system. (**a**), Representative frames of a movie acquired by the 2D CUP system [1] for ultrafast imaging of a laser pulse propagating in a 3D scattering medium. Only capable of 2D (x, y) ultrafast imaging, the previous CUP system gives inaccurate detection of time of arrival (ToA). (**b**), As in (**a**), but acquired by the SP-CUP system, which records the depth information and detects ToA correctly. (**c**) and (**d**) show the measured speeds of light (black solid lines) by tracing the centroids from the time-lapse laser pulse images captured by the previous 2D CUP system (**c**) and the SP-CUP system (**d**). The ground truth (red dashed lines) shows the distance-ToA relation using the known speed of light (i.e., 3×10^8 m s^{-1}). While the previous 2D CUP system, incapable of sensing depth, led to unacceptable deviation from the ground truth, the newly developed SP-CUP system showed high accuracy.

1 Liang, J. *et al.* Single-shot real-time video recording of photonic Mach cone induced by a scattered light pulse. *Sci. Adv.* **3**, e1601814 (2017).
2 Liang, J., Kohn, R. N., Becker, M. F. & Heinzen, D. J. Homogeneous one-dimensional optical lattice generation using a digital micromirror device-based high-precision beam shaper. *J Micro. Nanolithogr. MEMS MOEMS* **11**, 023002 (2012).
3 Liang, J., Wu, S.-Y., Kohn, R. N., Becker, M. F. & Heinzen, D. J. Grayscale laser image formation using a programmable binary mask. *Opt. Eng.* **51**, 108201 (2012).
4 Zhu, L. *et al.* Space-and intensity-constrained reconstruction for compressed ultrafast photography. *Optica* **3**, 694-697 (2016).
5 Heist, S., Dietrich, P., Landmann, M., Kühmstedt, P. & Notni, G. High-speed 3D shape measurement by GOBO projection of aperiodic sinusoidal fringes: a performance analysis in *Dimensional Optical Metrology and Inspection for Practical Applications VII.* 106670A (International Society for Optics and Photonics, 2018).
6 Goodman, J. W. *Introduction to Fourier optics*. (Roberts and Company Publishers, 2005).
7 Liang, J., Gao, L., Hai, P., Li, C. & Wang, L. V. Encrypted three-dimensional dynamic imaging using snapshot time-of-flight compressed ultrafast photography. *Sci. Rep.* **5**, 15504 (2015).
8 Gruev, V., Van der Spiegel, J. & Engheta, N. Dual-tier thin film polymer polarization imaging sensor. *Opt. Express* **18**, 19292-19303 (2010).
9 Liu, X., Liu, J., Jiang, C., Vetrone, F. & Liang, J. Single-shot compressed optical-streaking ultra-high-speed photography. *Opt. Lett.* **44**, 1387-1390 (2019).
10 Yang, C. *et al.* Single-Shot Receive-Only Ultrafast Electro-Optical Deflection Imaging. *Phys. Rev. Appl.* **13**, 024001 (2020).
11 Dabov, K., Foi, A., Katkovnik, V. & Egiazarian, K. Image denoising by sparse 3-D transform-domain collaborative filtering. *IEEE Trans. Image Process.* **16**, 2080-2095 (2007).
12 Yeola, S., Kuk, D. & Kim, K.-Y. Single-shot ultrafast imaging via spatiotemporal division of femtosecond laser pulses. *J. Opt. Soc. Am. B* **35**, 2822-2827 (2018).
13 Gosta, M. & Grgic, M. Accomplishments and challenges of computer stereo vision in *Proc. ELMAR.* 57-64 (IEEE).
14 Lazaros, N., Sirakoulis, G. C. & Gasteratos, A. Review of stereo vision algorithms: from software to hardware. *Int. J. Optomechatronics* **2**, 435-462 (2008).
15 Mathworks. *What Is Camera Calibration?*, https://www.mathworks.com/help/vision/ug/camera-calibration.html#buvr2qb-2
16 Mathworks. *Stereo Camera Calibration*, https://www.mathworks.com/help/vision/stereo-camera-calibration.html
17 Hirschmuller, H. Accurate and efficient stereo processing by semi-global matching and mutual information in *2005 IEEE Computer Society Conference on Computer Vision and Pattern Recognition (2005 CVPR).* 807-814 (IEEE, 2005).
18 Hirschmuller, H. Stereo processing by semiglobal matching and mutual information. *IEEE Trans. Pattern Anal. Mach. Intell.* **30**, 328-341 (2008).
19 Heikkila, J. & Silven, O. A four-step camera calibration procedure with implicit image correction in *1997 IEEE Computer Society Conference on Computer Vision and Pattern Recognition.* 1106-1112 (IEEE, 1997).
