# Supplementary material for: Single-shot stereo-polarimetric compressed ultrafast photography for light-speed observation of high-dimensional optical transients with picosecond resolution
Source: Nat Commun. 2020 Oct 16;11:5252. doi: 10.1038/s41467-020-19065-5 (PMC7567836; doi:10.1038/s41467-020-19065-5)
Supplement: Supplementary file 3 — Description of Additional Supplementary Files [file 41467_2020_19065_MOESM3_ESM.pdf]

Description of Additional Supplementary Files

Supplementary Movie 1. Reconstructed dynamics of a laser beam sweeping across five letters at 250 billion frames per second in plano-polarimetric ultrafast imaging. From left to right: reconstructed spatiotemporal dynamics using Views 1, 3, and 4 with 0° polarizers; reconstructed spatiotemporal dynamics using Views 2, 5, and 6 with 45° polarizers; reconstructed angle of linear polarization (AoLP, ψ); reconstructed first Stokes parameter (S_0).

Supplementary Movie 2. Reconstructed space-polarization-resolved dynamics of the laser-induced plasma on a silicon wafer, captured at 100 billion frames per second. Top left: reconstructed dynamics of the plasma plume shown with intensity contrast. The yellow dashed lines represent the plasma plume front. Top right: reconstructed dynamics of the plasma plume shown with polarization contrast. Bottom left: evolution of the averaged radius of the plume's front. Bottom right: evolution of the degree of linear polarization (DoLP) and angle of linear polarization (AoLP, ψ) averaged over the entire plume.

Supplementary Movie 3. Comparison of spatially resolved intensity dynamics of the laser-induced plasma on a silicon wafer. Left: the pump-probe sequence synthesized from ten repeated SP-CUP sequences. Right: the reconstructed sequence from one single SP-CUP acquisition.

Supplementary Movie 4. **Reconstructed dynamics of a laser beam sweeping across the three shapes at 100 billion frames per second in stereo-polarimetric ultrafast imaging.** Top left: reconstructed spatiotemporal dynamics using Views 2, 5, and 6 with 45° polarizers. Top right: reconstructed spatiotemporal dynamics using Views 1, 3, and 4 with 0° polarizers. Bottom left: four-dimensional visualization of the reconstructed angle of linear polarization (AoLP, ψ). Bottom right: four-dimensional visualization of the reconstructed first Stokes parameter (S_0).

Supplementary Movie 5. Reconstructed data in imaging an ultrashort laser pulse propagation in a three-dimensional (3D) scattering medium at 100 billion frames per second. I_{norm} represents light intensity normalized to the global maximum. Top left: reconstructed light intensity evolution in 3D space. Top middle: centroids of the pulse from reconstruction (red circles) and from the ground truth (cyan cross) in 3D space. Right: centroids of the pulse from reconstruction (red solid lines) and from the ground truth (cyan dashed lines) in three spatial dimensions. Bottom left: temporal intensity profiles at six 3D spatial locations labeled as magenta diamonds in the top middle section. Bottom middle: intensity cross sections

of the pulse at five representative frames. Bottom right: spatially-integrated total intensity and degree of linear polarization (DoLP) over the first 550 ps.
